# A retrospective study of adjuvant albumin-bound paclitaxel plus S-1 after D2 gastrectomy versus oxaliplatin plus S-1 in gastric cancer

**DOI:** 10.1038/s41598-024-65724-8

**Published:** 2024-07-02

**Authors:** Ning Li, Hui Wu, Xin Xu, Qinming Wei, Yongfeng Ding, Shan Liu, Jinqiong Wu, Yulong Zheng, Nong Xu, Yuan Gao, Haiping Jiang

**Affiliations:** The First Affiliated Hospital, Zhejiang University School of Medicine, Hangzhou, 310001 China

**Keywords:** Gastric cancer, Adjuvant chemotherapy, Albumin-bound paclitaxel, S-1, Disease free survival, Gastroenterology, Oncology

## Abstract

Adjuvant oxaliplatin plus S-1 (SOX) chemotherapy for gastric cancer (GC) after D2 gastrectomy has been proven effective. There has yet to be a study that evaluates adjuvant nanoparticle albumin-bound paclitaxel (nab-paclitaxel) plus S-1. In this single-center, retrospective study, GC patients after D2 gastrectomy received either nab-paclitaxel plus S-1 (AS group) or SOX group were recruited between January 2018 and December 2020 in The First Affiliated Hospital of Zhejiang University. Intravenous nab-paclitaxel 120 mg/m^2^ or 260 mg/m^2^ and oxaliplatin 130 mg/m^2^ were administered as eight 3 week cycle, especially in the AS and SOX group. Patients received S-1 twice daily with a dose of 40 mg/m^2^ in the two groups on days 1–14 of each cycle. The end points were disease-free survival (DFS) rate at 3 years and adverse events (AEs). There were 56 eligible patients, 28 in the AS group and 35 in the SOX group. The 3 year DFS rate was 78.0% in AS group versus 70.7% in SOX group (*p* = 0.46). Subgroup analysis showed that the patients with signet-ring positive in the AS group had a prolonged DFS compared with the SOX group (40.0 vs. 13.8 m, *p* = 0.02). The diffuse-type GC or low differentiation in the AS group was associated with numerically prolonged DFS compared with the SOX group, but the association was not statistically significant (*p* = 0.27 and *p* = 0.15 especially). Leukopenia (14.3%) were the most prevalent AEs in the AS group, while thrombocytopenia (28.5%) in the SOX group. Neutropenia (7.1% in AS group) and thrombocytopenia (22.8% in SOX group) were the most common grade 3 or 4 AEs. In this study analyzing past data, a tendency towards a greater 3 year DFS was observed when using AS regimen in signet-ring positive patients. AS group had fewer thrombocytopenia compared to SOX group. More studies should be conducted with larger sample sizes.

## Introduction

Worldwide, there are more than one million fresh instances of gastric cancer (GC), placing it fifth in terms of occurrence and fourth in terms of fatality^1^. In 2020, it was estimated that there were 769,000 deaths (equivalent to one in every 13 deaths). There is a two-fold increase in male rates compared to female rates. There is a serious burden of GC in China due to its high incidence. According to the latest data^2^, GC ranked as the third most prevalent cancer and the third leading cause of cancer-related deaths in China in 2022.

Surgery represents the primary treatment option for managing early-stage and locally advanced GC. Recurrence rates following surgery are high, with approximately 40% of patients experiencing relapse within two years after surgery. GC spreads through the lymphatic system, bloodstream, and peritoneum early in the disease process^3^. Different adjuvant treatments have been investigated for decades to enhance post-surgical relapse control. Several large phase III clinical studies have demonstrated that adjuvant chemotherapy after D2 radical resection can improve survival outcomes in GC patients^4–6^. The trial showed that S-1 monotherapy as adjuvant chemotherapy provided a survival advantage for stage II-III GC^4^. Based on the CLASSIC study, oxaliplatin combined with capecitabine (CapOx) was recommended as a postoperative therapy option, resulting in a 3 year disease-free survival (DFS) rate of 74% for those treated with chemotherapy and surgery and 59% for those treated with surgery alone^5^. After surgery, various combinations of anticancer medications were investigated for their viability, considering that S-1, which is commonly used in Asia to treat GC, has fewer negative impacts compared to fluorouracil. The RESLOVE study found that the adjuvant CapOx group had a 3 year DFS rate of 51.1%, while the adjuvant SOX group had a rate of 56.5%, suggesting that there was no significant difference between the two groups in terms of non-inferiority^7^. Furthermore, the JACCRO GC07 trial exhibited a higher 3 year DFS rate (66% vs. 50%, *p* < 0.05) when utilizing S-1 in combination with docetaxel as opposed to S-1 alone^6^.

Stomach and gastroesophageal cancers have demonstrated advantages in treatment when combined with chemotherapy and the use of immune checkpoint inhibitors (ICIs) like programmed cell death receptor-1 (PD-1) and programmed cell death-ligand 1 (PD-L1) inhibitors^8–10^. KEYNOTE-585, an evaluation of pre-operative and postoperative ICI treatment for locally advanced GC have demonstrated that the use of pembrolizumab, compared to a placebo, resulted in an enhancement of the pathological complete response^11^. However, this improvement did not lead to a significant increase in DFS among the patients. In yet another phase 3 trial in Asian patients with pathological stage III gastroesophageal junction adenocarcinomas, nivolumab plus chemotherapy was used after surgery, also did not display any improvement in DFS^12^. The patients after D2 gastrectomy always has poor nutritional status, which is closely related to survival outcomes with anti-cancer treatments. A meta-analysis has reported that albumin levels may be a prognostic biomarker in advanced cancer patients treated with ICIs^13^.On the other hand, immortal time bias^14^ and hypertransaminasemia^15^ should also be further studied when treated with ICIs.

A neoadjuvant approach for GC has been found to be more effective than surgery alone at reducing tumor burden, assessing tumor response, and improving DFS by the large phase III clinical studies such as FLOT4^16,17^, PRODIGY^18^ and RESOLVE studies^7^. Recently, a statistically significant shorter DFS (HR: 3.30, 95%CI 1.50–7.35, *p* = 0.003) were observed for patients with signet-ring cell positive who received perioperative treatment vs. those who received surgery followed by adjuvant chemotherapy^19^. As a retrospective study, it indicated that new postoperative protocols for special types of GC such as signet-ring cell positive are still unsatisfactory.

Paclitaxel in combination with an oral fluoropyrimidine is a potential treatment option for resected gastric cancers that are at a high risk of peritoneal recurrence. For the first time, the SAMIT study compared the effectiveness of sequential treatment with monotherapy, specifically paclitaxel followed by either tegafur and uracil (UFT) or S-1 after paclitaxel^20^. Although the DFS was not improved with sequential treatment, it was found to be superior to monotherapy in stage IIIb patients, despite not being significant. A current study is in progress to evaluate the efficacy of POF (paclitaxel/oxaliplatin/5-fluorouracil/leucovorin) and SOX/CapOx/FOLFOX in comparison for stage III GCs following surgical intervention^21^.

The use of albumin-bound paclitaxel (nab-paclitaxel) provides a benefit compared to solvent-based paclitaxel by enabling the administration of a greater amount of paclitaxel to tumors while decreasing the incidence of severe adverse reactions. A prior investigation confirmed the safety and efficacy of the combination of nab-paclitaxel and S-1 (AS) in individuals diagnosed with advanced GC^22,23^. The NORDICA study also confirmed that the recommended phase 2 dose (RP2D) of nab-paclitaxel as adjuvant chemotherapy for diffuse-type gastric cancer (DGC) is 260 mg/m^2^, and the AS regimen showed tolerable adverse events in stage III DGC^24^. In another research, the effectiveness of additional AS therapy was assessed in comparison to CapOx following D2 gastrectomy in individuals diagnosed with stage III GC^25^. Up until now, there have been no studies that have compared the AS treatment with the suggested SOX treatment in individuals diagnosed with stage II–III GC. Our center has recently conducted the administration of AS as an additional chemotherapy for GC patients with DGC type, low differentiation and signet-ring cell carcinoma. This retrospective study was conducted to compare the AS regimen with SOX treatment. The primary goal of this study is to compare the 3 year DFS and adverse events (AEs) rate.

## Patients and methods

### Study design and participants

This is a single center, retrospective study carried out at The First Affiliated Hospital of Zhejiang University. After undergoing D2 radical resection, the study identified individuals diagnosed with stage II–III gastric adenocarcinoma (gastroesophageal junction) who successfully achieved R0 resection. The patients who received either a combination of nab-paclitaxel and S-1 (referred to as the AS group) or oxaliplatin with S-1 (referred to as the SOX group) from January 2018 to December 2020 were enrolled. The assignment of patients to the AS or SOX group was not done at random. This is a better reflection of real world treatment because the doctor’s decisions were based on the patient's physical score, economic level, expectation for efficacy and requirements for side effects. This study was approved by the Clinical Research Ethics Committee of the First Affiliated Hospital, Zhejiang University School of Medicine (approval number: IIT20240193A).The study has been performed in accordance with the Declaration of Helsinki.

### Treatment and outcome

Patients treated with nab-paclitaxel combined with S-1were assigned to the AS group, whereas those receiving oxaliplatin and S-1 were assigned to the SOX group. Orally, S-1 with a dose of 40 mg/m^2^ was given twice daily from days 1–14. Intravenous infusion of nab-paclitaxel was administered at a dosage of 120 mg/m^2^ on days 1 and 8, or 260 mg/m^2^ on day 1 during each cycle. 130 mg/m^2^ of intravenous oxaliplatin was administered on day 1. The main endpoint was 3-year DFS and AE. DFS was characterized as the duration starting from surgical procedure until the occurrence of primary cancer recurrence, fresh gastric cancer, distant metastases (evaluated by each researcher), or demise from any reason, whichever happened earlier. The overall survival rate (OS) of a patient with cancer was determined as the duration from the date of diagnosis until the occurrence of death, irrespective of the cause. R0 surgery refers to the surgical procedure where there is a thorough removal of all tumors, leaving no visible or invisible traces of cancerous cells. AEs were assessed using version 5.0 of the National Cancer Institute Common Terminology Criteria for Adverse Events (CTCAE).

### Statistical analysis

Frequency and percentage were used to describe qualitative variables, and median and range were used to describe continuous variables. DFS and OS were explained using a median and 95% confidence interval (CI) according to the Kaplan–Meier approach. Survival analyses were performed to compare groups using the logrank test. All tests were considered statistically significant if the p-value was less than 0.05.

### Ethical approval

This study was approved by the Clinical Research Ethics Committee of the First Affiliated Hospital, Zhejiang University School of Medicine (approval number: IIT20240193A). Due to the retrospective study design with no intervention for enrolled patients, the Clinical Research Ethics Committee of the First Affiliated Hospital, Zhejiang University School of Medicine waived informed consent.

## Results

### Patient characteristics

**Figure 1:**
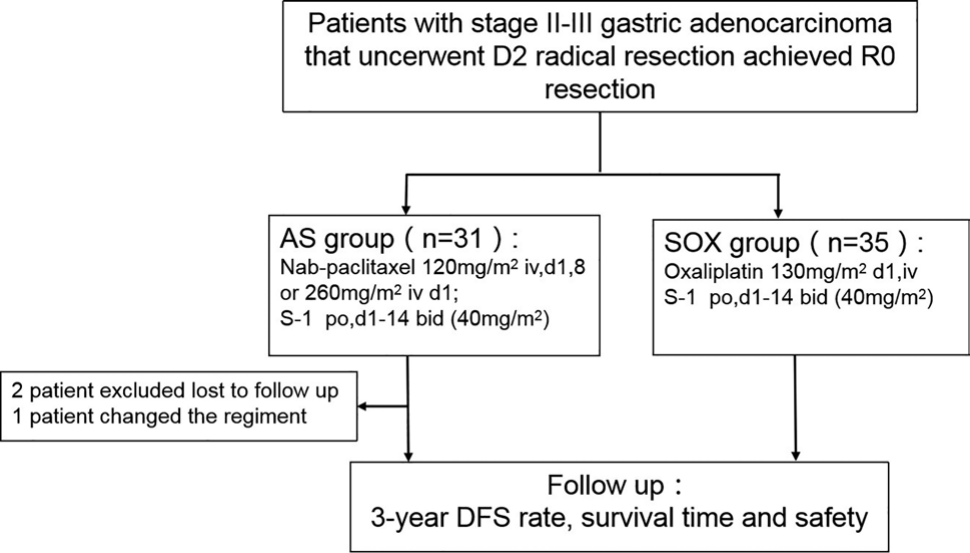
Trial Flowchart.

There were 66 GC patients eligible to participate in this study between January 2018 and December 2020. Figure 1 illustrates the trial profile. Twenty eight patients administered nab-paclitaxel combined with S-1, and thirty five patients received oxaplatin plus S-1. The median age was 57.5 years (29–73 years) in AS group, including 15 men (53.6%) and 13 women (46.4%). In SOX group, the median age was 62.0 years (35–80 years), with 25 men (71.4%) and 10 women (28.6%). Table 1 provides a summary of the baseline characteristics of the patients.

**Table 1:** Baseline demographic and patient characteristics.

Characteristic	AS group^a^ (n = 28)	SOX group^b^ (n = 35)
Age (years)		
Median (range)	57.5 (29–73)	62.0 (35–80)
Genda, n (%)		
Male	15 (53.6)	25 (71.4)
Female	13 (46.4)	10 (28.6)
ECOG performance status, n (%)		
0	22 (78.6)	29 (82.9)
1	6 (21.4)	6 (17.1)
Histological type, n (%)		
Adenocarcinoma	14 (50.0)	29 (82.9)
Signet cell carcinoma	14 (50.0)	6 (17.1)
Grade, n (%)		
Medium differentiation	6 (21.4)	25 (71.4)
Low differentiation	22 (78.6)	10 (28.6)
Lauran’s classification, n. (%)		
Intestinal	2 (7.1)	16 (45.7)
Diffuse	17 (60.7)	5 (14.3)
Mixed	9 (32.1)	13 (37.1)
Unknown or unclassifiable	0	1 (2.9)
Pathological stage, n (%)		
II	13 (46.4)	17 (48.6)
IIIA	6 (21.4)	11 (31.4)
IIIB	7 (25.0)	4 (11.4)
IIIC	2 (7.1)	3 (8.6)
Pathological T stage, n (%)		
T1	4 (14.3)	3 (8.6)
T2	9 (32.1)	9 (25.7)
T3	11 (39.3)	11 (31.4)
T4	4 (14.3)	12 (34.3)
Pathological N stage, n (%)		
N0	1 (3.6)	4 (11.4)
N1	5 (17.9)	10 (28.6)
N2	8 (28.6)	12 (34.3)
N3	14 (50.0)	9 (25.7)

### Efficacy and survival

**Figure 2:**
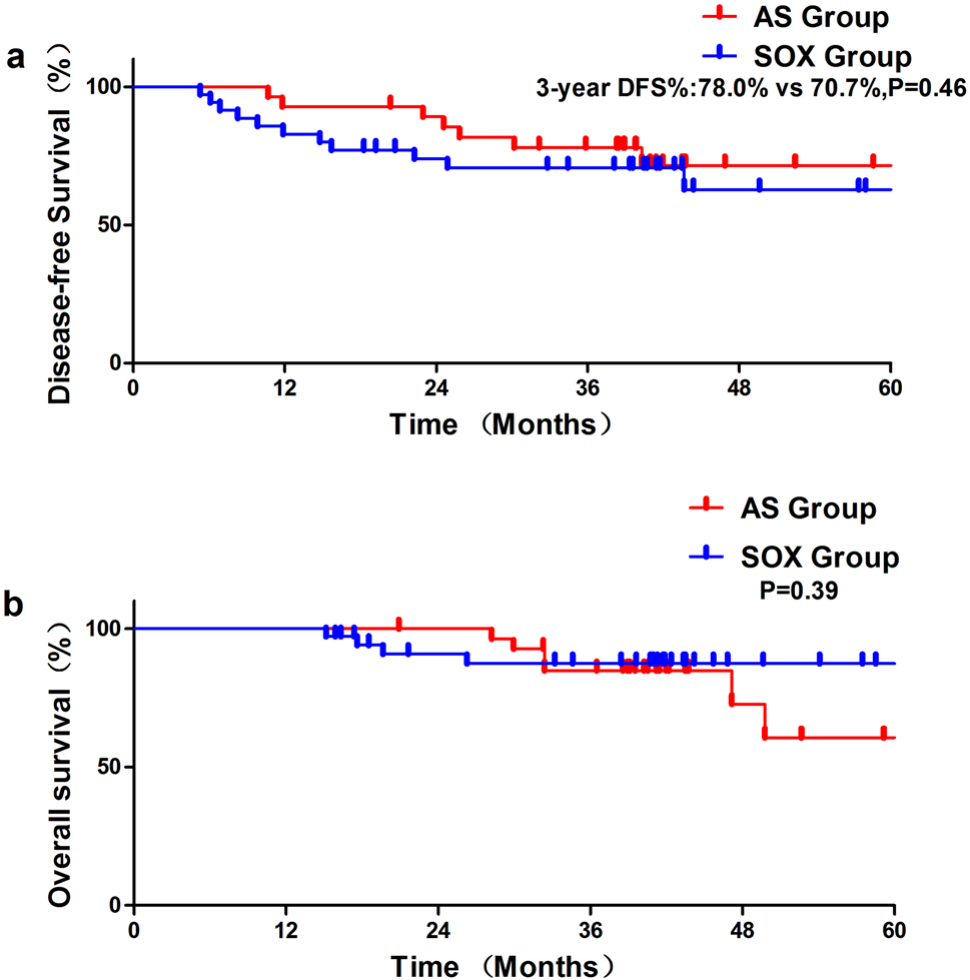
3 year disease-free survival (**a**) and overall survival (**b**) of the AS and SOX group.

To date 30 May 2024, the median duration of follow-up was 44.9 months (20.9–65.1) in the AS group and 45.3 months (15.9–78.4) in the SOX group. 7 patients (20%) in the AS group had relapsed, or new occurrences of GC, 5 of the cases were DGC and 2 was mixed type. While 11 patients (31.4%) had recurrence in the SOX group with 4 DGC, 3 mixed type and 4 intestinal type. The 3-year DFS rate was 78.0% in AS group versus 70.7% in SOX group (*p* = 0.46). There is an early separation between the two AS and SOX study groups according to Kaplan–Meier curves for DFS (Fig. 2a). Compared to the SOX group, the AS group died more frequently (6/21.4%) than the SOX group (4/11.4%). The AS group had a 3 year OS rate of 84.8%, while the SOX group had a rate of 87.4% (Fig. 2b). One patient in the AS group died because of Amyotrophic Lateral Sclerosis.

**Figure 3:**
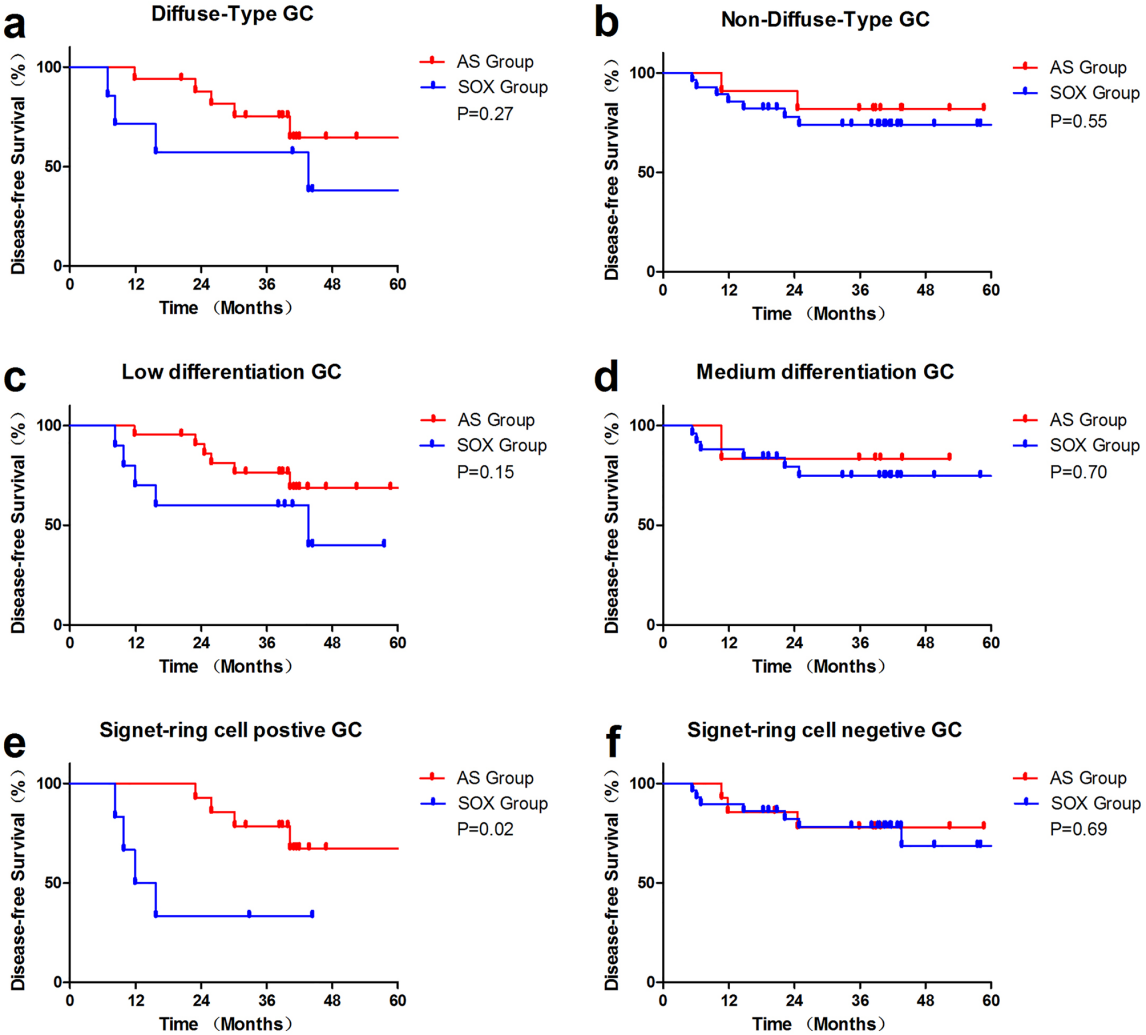
3 year disease-free survival for the subgroups of the AS and SOX group. (**a**) Diffuse-Type GC, (**b**) Non-Diffuse-Type GC, (**c**) Low differentiation GC, (**d**) Middle differentiation GC, (**e**) Signet-ring positive GC, (**f**) Signet-ring negative GC.

Subgroup analysis showed there were 17 DGC in the AS group, the median 3-DFS was 48.9 m, with a 3-DFS rate of 75.3% (Fig. 3a). 21 patients in the AS group were low differentiated, with a median DFS of 50.0 m and a 3-DFS rate of 76.3% (Fig. 3c). The patients with DGC or low differentiation in the AS group was associated with numerically prolonged DFS compared with the SOX group, but the association was not statistically significant (*p* = 0.27 and *P* = 0.15 especially). There were 14 signet-ring positive patients in the AS group, which had a prolonged DFS compared with the SOX group (40.0 vs. 13.8 m, *p* = 0.02, Fig. 3e). While in the patients with non-DGC (Fig. 3b), medium differentiation (Fig. 3d) and signet-ring negative (Fig. 3f) gastric cancer, the DFS of SPA group and SOX group were approximate with no statistical significance.

### Safety

The AEs in AS group most commonly found were leukopenia (14.3%), neutropenia (10.7%) and peripheral neuropathy (10.7%) (Table 2), while in the SOX group were neutropenia (14.3%) and thrombocytopenia (28.5%). Neutropenia (7.1%) was the prevailing grade 3 or 4 AE in the AS group, whereas thrombocytopenia (22.8%) was more frequent in the SOX group. No instances of peripheral neuropathy, specifically in Grade 3 or 4, were detected in relation to nab-paclitaxel and oxaliplatin.

**Table 2:** The major adverse events (AEs).

	Grades 1–2		Grade 3–4	
	AS group^a^(n = 28)	SOX group^b^(n = 35)	AS group^a^(n = 28)	SOX group^b^(n = 35)
Anemia	1 (3.6)	0	0	0
Leukopenia	4 (14.3)	1 (2.9)	0	1 (2.9)
Neutropenia	1 (3.6)	1 (2.9)	2 (7.1)	4 (11.4)
Thrombocytopenia	1 (3.6)	2 (5.7)	0	8 (22.8)
Peripheral neuropathy	3 (10.7)	0	0	0
Nausea	0	0	0	2 (5.7)
Increased glutamic oxalacetic transaminase	0	0	0	1 (2.9)

Data are presented as n (%).

Adverse events were graded according to the National Cancer Institute’s Common Terminology Criteria for Adverse Events, version 5.0.

## Discussion

In this retrospective study, conducted at a single center, individuals diagnosed with stage II–III gastric cancer following D2 gastrectomy exhibited marginal enhancement in 3-year DFS (78.0%) when treated with the AS regimen, as opposed to the SOX treatment (70.1%), but not significantly so. The 3-year DFS of SOX regimen was consistent with the reported clinical trials. CLASSIC study reported a 3-year DFS of 74% with adjuvant CapOx regimen^5^, while the 3-year DFS was 56.5% and 66% in RESOLVE study with SOX treatment^7^ and in JACCRO GC07 trial with S-1 plus docetaxel^6^, respectively.

Lauren classifies GC into intestinal type (IGC), diffuse type (DGC) and mixed type. IGC are cancers that are highly differentiated, whereas DGC are cancers that are characterized by poor differentiation and have worse prognoses. More significantly, in our study, the AS group has a higher percentage of DGC (60.7% vs. 14.3%)and low differentiation (78.6% vs. 28.6%) than the SOX group. The adjuvant SOX group in RESOLVE study included 51% IGC^7^, and only 7.1% in the AS group of our study. Otherwise, the AS group contributed with 50.0% signet-ring cell carcinoma, which all indicated lower survival. A worse prognosis is therefore expected for the AS group in our study. A real-life study found that IGC and DGC might be different in their sensitivity to chemotherapeutic agents, and the role of oxaliplatin as an adjuvant treatment among 580 participants was analyzed after D2 gastrectomy^26^. The median DFS for DGC and IGC were 21.2 and 32.2 months, respectively, with a HR of 1.56 and p-value less than 0.001. So far, there has been only one reported phase I trial that utilized the adjuvant AS regimen for stage III DGC, and there is no available report on the 3-year DFS. While in our study, there were 17 DGC in the AS group, the median 3-year DFS was 48.9 m, with a 3-DFS rate of 75.3%. Signet-ring positive GC was reported with a poor median DFS of 26.3 months^19^. There were 14 signet-ring positive patients in AS group of our study, which had a prolonged DFS compared with the SOX group (40.0 vs 13.8 m, *p* = 0.02). Therefore, our research suggests that nab-paclitaxel in combination with S-1 could be a potentially beneficial adjuvant treatment option for gastric cancer, particularly for signet-ring positive GC and DGC.

Sasaki et al. had found that nab-paclitaxel (administered at a dose of 260 mg/m^2^ on days 1 per 3 weeks demonstrated efficacy and good tolerability as a second-line chemotherapy option for advanced GCs that had been treated before^27^. Previously, the dose of nab-paclitaxel plus S-1 was escalated in Chinese patients^23^. The highest acceptable amount of nab-paclitaxel was not achieved at any of the three dosage levels (80 mg/m^2^, 100 mg/m^2^, or 120 mg/m^2^) when administered on days 1 and 8, q3w. According to the NORDICA study, the combination of S-1 and nab-paclitaxel showed tolerability at dose levels of up to 260 mg/m^2^^24^. In our research, nab-paclitaxel was administered either at a dosage of 120 mg/m^2^ on days 1 and 8 every 3 weeks or at a dosage of 260 mg/m^2^ on day 1 every 3 weeks. The most commonly included AEs with any grade in the AS group was leukopenia (14.3%), while in the SOX group were neutropenia (14.3%) and thrombocytopenia (28.5%). The predominant grade 3 or 4 AE in the AS group was neutropenia (7%), whereas thrombocytopenia (22.8%) was more frequent in the SOX group. In the previous studies, thrombocytopenia occured in 34%^5^ and 21%^7^ in patients treated with adjuvant CapOx and SOX regimen, especially. The incidence of thrombocytopenia in the SOX group was 35.1%, while in the AS group was 15.1% in the first line treatment of advanced GC, and this may be related to oxaliplatin^28^. These were also consistent with our findings. As we all know, thrombocytopenia could’t be treated more effectively and quickly than neutropenia. From the perspective of adverse reactions, AS program may be a better choice as adjuvant therapy. No instances of peripheral neuropathy, specifically in Grade 3 or 4, were detected in relation to nab-paclitaxel and oxaliplatin.

## Conclusions

In conclusion, this retrospective cohort study provides the first information on long-term outcomes after adjuvant nab-paclitaxel versus oxaliplatin combined with S-1 after D2 gastric resection for stage II-III GCs. A trend with higher DFS was identified in AS group, especially for signet-ring positive GC. Compared with SOX group, AS group had fewer serious thrombocytopenia. The need for further randomized controlled studies in larger cohorts needs to be addressed.

## Data Availability

The datasets used and/or analysed during the current study available from the corresponding author on reasonable request.
